# Recent Advances on Building Information Modeling

**DOI:** 10.1155/2015/786598

**Published:** 2015-05-21

**Authors:** Yu-Shen Liu, Heng Li, Haijiang Li, Pieter Pauwels, Jakob Beetz

**Affiliations:** ^1^School of Software, Tsinghua University, Beijing 100084, China; ^2^Department of Building and Real Estate, The Hong Kong Polytechnic University, Hong Kong; ^3^BRE Institute of Sustainable Engineering, Engineering School, Cardiff University, UK; ^4^Department of Architecture and Urban Planning, Ghent University, 9000 Ghent, Belgium; ^5^Department of the Built Environment, Eindhoven University of Technology, Netherlands

Building information modeling (BIM) technology has been receiving an increasing attention in the architecture, engineering, and construction (AEC) industry. Unlike traditional computer aided design (CAD) technology, BIM technology allows storing both geometric information and rich semantic information of building models, as well as their relationships, to support lifecycle data sharing. In terms of information technology (IT) adoption, BIM is a new trend in the AEC industry. However, BIM still suffers from lack of fundamental research, such as the representation, exchange, and interoperability of information. In addition, research on various BIM applications is also very prospective. These trends provide new challenges and opportunities for researchers.

This special issue aims to bring researchers from academia and industry together to report and explore new methodologies and applications in BIM and review the latest progress in this field. Out of about forty submissions, five research articles have been selected and included in this special issue due to their good quality and relevance to the theme. The selected papers address various aspects, including a translation approach between BIM and building energy modeling, a practical method with application to facility management (FM) using 2D barcodes and BIM technologies, a BIM-based virtual environment for fire emergency evacuation, a BIM data application to building performance simulation software for the early phases of building design, and a BIM-based approach in construction to support the construction procurement process.

The exchange of data between building design representations and energy simulation representation has been a major challenge in the design process, resulting in the fact that building energy performance simulation is often omitted from the process. The translation process is labor intensive, error-prone, and cumbersome. Although tools have been developed to support the generation of an energy model from a design model, disconnections still exist between the various models. In practice, many of the problems derive from building energy simulation tools that fail to take advantage of object-oriented programming (OOP) and do not easily allow for mapping from an object-oriented design model. To improve and enhance the model translation effectiveness, W. Jeong et al., in their paper titled “Translating Building Information Modeling to Building Energy Modeling Using Model View Definition”, present a translation approach to translate between BIM and building energy modeling (BEM) that uses Modelica, an object-oriented declarative, equation-based simulation environment. A tool, named BIM2BEM, has been developed using a data modeling method to enable seamless model translations of building geometry, materials, and topology. Using data modeling, the authors created a model view definition (MVD) consisting of a process model and a class diagram. The process model demonstrates object mapping between BIM and Modelica-based BEM (ModelicaBEM) and facilitates the definition of required information during model translations. The class diagram represents the information and object relationships to produce a class package intermediate between the BIM and BEM. The implementation of the intermediate class package enables system interface (Revit2Modelica) development for automatic BIM data translation into ModelicaBEM. In order to demonstrate and validate the approach, simulation result comparisons have been conducted via several test cases.

Facility management (FM) has become an important topic in research on the operation and maintenance phase. Effectively managing a facility is extremely difficult owing to the variety of environments. One of the difficulties is the performance of two-dimensional (2D) graphics when depicting facilities. BIM uses precise geometry and relevant data to support the facilities depicted in 3D object-oriented CAD. To address this issue, Y.-C. Lin et al., in their paper titled “Developing Mobile BIM/2D Barcode-Based Automated Facility Management System,” propose a practical method with application to FM that uses an integrated 2D barcode and the BIM approach. Using a 2D barcode and BIM technologies, this study proposes a mobile automated BIM-based facility management (BIMFM) system for FM staff in the operation and maintenance phase. The mobile automated BIMFM system is then applied in a selected case study of a commercial building project in Taiwan to verify the proposed methodology and demonstrate its effectiveness in practice. The combined results demonstrate that a BIMFM-like system can be an effective mobile automated FM tool. The advantage of the mobile automated BIMFM system lies not only in improving efficiency for the FM staff but also in facilitating FM updates and transfers back into the BIM environment.

Recent building emergency management research has highlighted the need for the effective utilization of dynamically changing building information. BIM can play a significant role in this process due to its comprehensive and standardized data format and integrated process. To address this issue, B. Wang et al., in their paper titled “BIM Based Virtual Environment for Fire Emergency Evacuation,” introduced a BIM-based virtual environment supported by virtual reality (VR) and a game engine to address several key issues for building emergency management, for example, timely two-way information updating and better emergency awareness training. The focus of this paper lies on how to utilize BIM as a comprehensive building information provider to work with VR technologies to build an adaptable immersive serious game environment to provide real-time fire evacuation guidance. The innovation lies in the seamless integration between BIM and a VR environment, thereby aiming at practical problem solving by leveraging state-of-the-art computing technologies. The system has been tested for its robustness and functionality against the development requirements, and the results show promising potential to support more effective emergency management.

Data mining techniques are not often used in combination with building information modeling (BIM) technology. Nevertheless, applying data mining techniques on a database of BIM models could provide valuable insights in key design patterns implicitly present in these BIM models. The architectural designer would then be able to use previous data from existing building projects as default values in building performance simulation software for the early phases of building design. K. Hiyama, in the paper titled “Assigning Robust Default Values in Building Performance Simulation Software for Improved Decision-Making in the Initial Stages of Building Design,” proposed a method to minimize the magnitude of variation in these default values in subsequent design stages. This approach maintains the accuracy of the simulation results in the initial stages of building design. In this study, an argument is presented to demonstrate the significance of the new method. The variation in the ideal default values for different building design conditions is assessed first. Next, the influence of each condition on these variations is investigated. The space depth is found to have a large impact on the ideal default value of the window-to-wall ratio, whereas the window orientation has little impact. In addition, the presence or absence of lighting control and natural ventilation has a significant influence on the ideal default value. These effects can be used to identify the types of building conditions that should be considered to determine ideal default values for a new building design project.

Finally, A. A. Costa and A. Grilo, in their paper titled “BIM-Based E-Procurement: An Innovative Approach to Construction E-Procurement,” describe a particular BIM application with an approach to e-procurement in construction, which uses BIM to support the construction procurement process. The result is an integrated and electronic instrument connected to a rich knowledge base capable of advanced operations and able to strengthen transaction relationships and collaboration throughout the supply chain. The BIM-based e-procurement prototype has been developed using distinct existing electronic solutions and an IFC server and was tested in a pilot case study, which supported further discussions of the results of the research.

*Yu-Shen Liu* *Yu-Shen Liu* *Heng Li* *Heng Li* *Haijiang Li* *Haijiang Li* *Pieter Pauwels* *Pieter Pauwels* *Jakob Beetz* *Jakob Beetz*

